# Pulmonary Manifestations in Von Recklinghausen’s Disease: A Rare Presentation

**DOI:** 10.7759/cureus.23365

**Published:** 2022-03-21

**Authors:** Luke R Leggett, Francin Alexis, Nikita Agarwal, Zachary Bakhtin, Banu Farabi

**Affiliations:** 1 Medicine, St. Peter's University Hospital, New Brunswick, USA; 2 Internal Medicine, St. Peter's University Hospital, New Brunswick, USA

**Keywords:** cafe-au-lait spots, preventative medicine, von recklinghausen disease, neurofibromas, internal medicine, dermatology, pulmonology, genetics, neurofibromatosis associated diffuse lung disease, neurofibromatosis type 1 (nf-1)

## Abstract

Neurofibromatosis type 1 is a genetic disease that leads to a specific collection of symptoms. Most patients over time develop cutaneous manifestations, which include neurofibromas, freckling, or even cafe-au-lait spots. In general, patients with NF1 have a shorter life expectancy than non-affected individuals. This report aims to present our patient with NF1 and one of its rare manifestations, neurofibromatosis with diffuse lung disease. Hopefully, by describing this case and our patient's condition, it will serve as a resource to those treating similar patients.

## Introduction

Neurofibromatosis (NF) type 1, or von Recklinghausen disease, is an autosomal dominant condition due to a mutation in the NF1 gene on Chromosome 17. This condition is due to a defect in the neurofibromin protein, whose expression is found high in the lungs, liver, placenta, kidneys, and skeletal muscle. Neurofibromin is a known GTPase-activating protein whose function is to activate and control the RAS/MAPK pathway. This disease is known to affect approximately one out of 3,000 live births [1]. These patients have a variety of phenotypic expressions due to a high level of genetic variabilities, such as lisch nodules, cafe-au-lait spots, and cutaneous neurofibromas. One particularly rare form of this disease is the pulmonary involvement known as NF with diffuse lung disease (NF-DLD). There have been few studies on NF-DLD. Due to the rarity of the disease, the overall prevalence, and clinical presentation remains unclear. We present a 70-year-old female patient with a rare form of NF-1 showing progressive lung involvement on serial CT scans. We aim to increase awareness of this rare disease along with CT and clinical images.

## Case presentation

**Figure 1:**
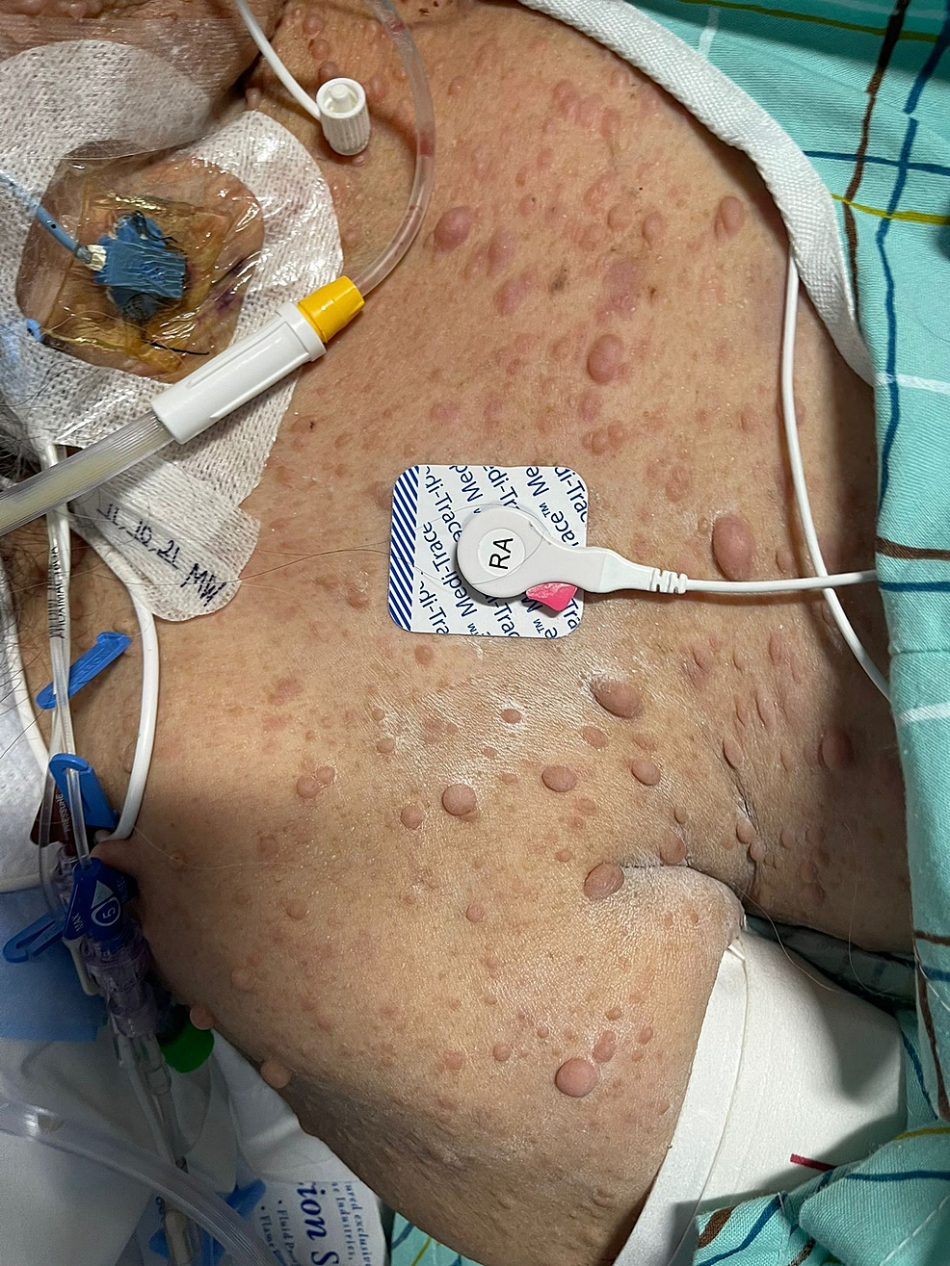
Our patient's anterior chest wall, which shows numerous cutaneous neurofibromas, freckling, and several cafe-au-lait spots.

**Figure 2:**
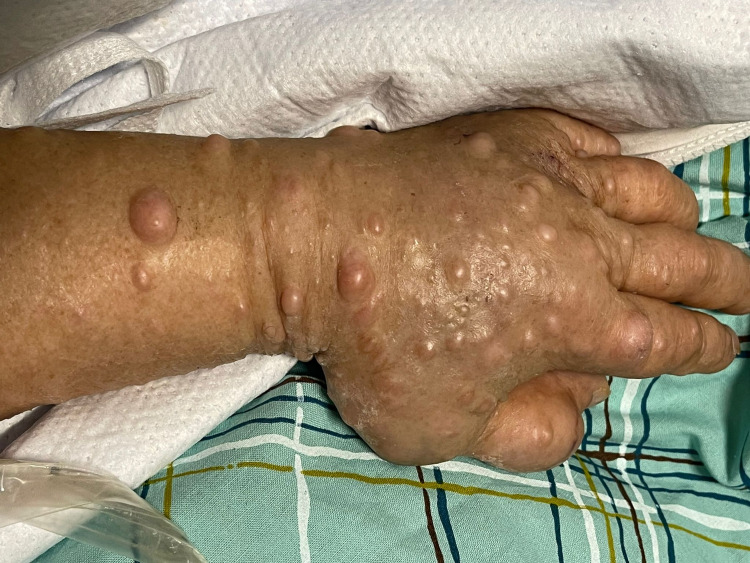
Patient's left wrist and hand, showing extensive neurofibromas.

Our patient was initially diagnosed with NF-1 at the age of 16. The patient at the time was the only person in her family with the phenotypical presentation of NF1. From questioning the patient, she did not have any relatives with NF1 either [2]. She first developed the cutaneous manifestations of NF-1, such as skin freckling, cafe-au-lait spots, and cutaneous neurofibromas. Eventually, the patient’s skin was covered by the cutaneous neurofibromas (Figures 1, 2). Several decades into the progression of NF-1, our patient was seen in the hospital for dyspnea. During this admission, she was mildly hypoxic, and a pulmonary workup was initiated. After several tests and a CT scan of her lungs, she was diagnosed with cystic pulmonary disease. On CT imaging, the disease was initially localized to the periphery of both her lungs. Upon lung biopsy several months later, the patient’s disease was confirmed to be NF-DLD. Over the course of seven years, our patient's lungs were slowly replaced with cystic nodules. Interestingly enough, the patient's relatively stable cystic disease progressed to a diffuse cystic lung disease seen in all lung fields. This eventually led to a fatal course within the last three months. She required frequent hospitalization and follow-up in the ICU due to treatment-resistant hypoxia. Upon our patient's last admission, she was seen in acute respiratory distress placed on BiPAP in the emergency department. Due to persistent hypoxia on BiPAP, she eventually required intubation and mechanical ventilation. The patient's last CT scan revealed extensive, diffuse, and bilateral cystic lesions with confluent alveolar infiltration. Due to the complicated nature of her disease, she has since passed.

## Discussion

NF-DLD is generally an incidental finding on x-ray which warrants further investigation using a high-resolution CT scan (HRCT) [3]. A typical HRCT scan will show a thickened lung parenchyma with well-defined borders around the cyst or bullae in the upper lobe, which can be differentiated from an emphysematous lung which often demonstrates an ill-defined border. The prevalence remains unclear; however, it is reported in 10%-20% of adults with the disease [4,5].

**Figure 3:**
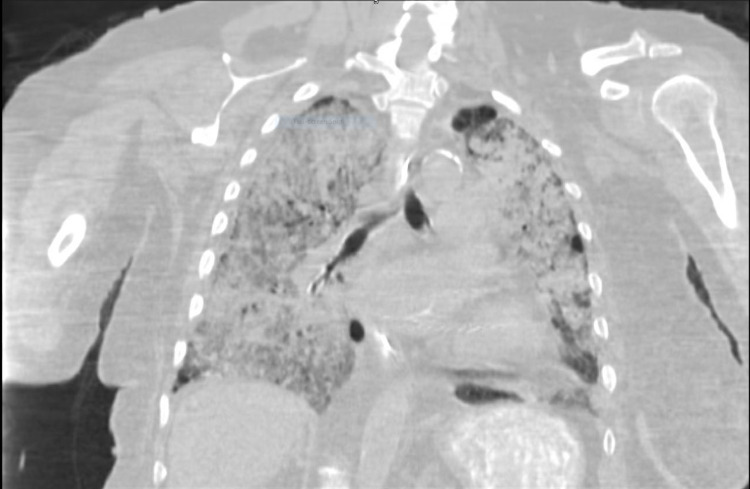
HRCT scan of our patient's chest showing the diffuse lung disease and peripheral nodules. HRCT scan = High-resolution CT scan

Clinically, these patients may initially present with signs of airway obstruction such as cough, wheezing, and dyspnea. Pulmonary function testing often shows an obstructive or restrictive pattern with a decreased DLCO. These patients can present with recurrent episodes of pneumonia-like symptoms, leading to the misdiagnosis of their real underlying etiology, causing these individuals to receive improper treatment. Clinicians without knowledge of possible lung involvement in individuals with NF-1 may overlook this initial presentation of NF-DLD. Delayed diagnosis can lead to many complications, such as spontaneous pneumothorax due to a ruptured subpleural bleb, vascular remodeling leading to pulmonary hypertension, and chronic respiratory failure (Figure 3) [6,7].

Patients diagnosed with NF-1 have a known decrease in life expectancy by about 15 years [8]. Presently there are no definitive treatments to any variations of NF-1, making it difficult to manage. Currently, NF-DLD can be managed through medical intervention and symptomatic relief in order to slow the progression [9]. Therefore, it may be essential to set protocols to have these individuals routinely screened for secondary prevention and ensure they receive appropriate care. The main goal for clinicians should be focused on limiting the onset of possible complications. Doing so will certainly improve the patient’s quality of life, further prolonging the individual life expectancy.

## Conclusions

The importance of this report is to show that NF-DLD is not like NF1 alone and to shed light on some of the more malicious processes that can occur. Understanding NF-DLD itself and being aware of the patient's possible clinical course is critical. This is a rare disease. Therefore, no protocol or standard treatments are available. It is vital that primary physicians of these patients routinely see these patients and educate them on reasons for seeking early intervention. Physicians should request that their patients with NF1 be seen annually for surveillance. Those patients who show signs of early pulmonary involvement should undergo High-resolution CT imaging for further workup.
